# Effects of a Resident Yeast from the Honeybee Gut on Immunity, Microbiota, and *Nosema* Disease

**DOI:** 10.3390/insects10090296

**Published:** 2019-09-13

**Authors:** James P. Tauber, Vy Nguyen, Dawn Lopez, Jay D. Evans

**Affiliations:** Bee Research Laboratory, Beltsville Agricultural Research Center, US Department of Agriculture, Beltsville, MD 20705, USA; nguyenvy201@gmail.com (V.N.); Dawn.Lopez@ars.usda.gov (D.L.)

**Keywords:** honeybee, yeasts, health, microbes, microbiota

## Abstract

The western honeybee (*Apis mellifera*) has a core bacterial microbiota that is well described and important for health. Honeybees also host a yeast community that is poorly understood with respect to host nutrition and immunity, and also the symbiotic bacterial microbiota. In this work, we present two studies focusing on the consequences of dysbiosis when honeybees were control-fed a yeast that was isolated from a honeybee midgut, *Wickerhamomyces anomalus*. Yeast augmentation for bees with developed microbiota appeared immunomodulatory (lowered immunity and hormone-related gene expression) and affected the microbial community, while yeast augmentation for newly emerged bees without an established bacterial background did not lead to decreased immunity— and hormone—related gene expression. In newly emerged bees that had a naturally occurring baseline level of *W. anomalus*, we observed that the addition of *N. ceranae* led to a decrease in yeast levels. Overall, we show that yeasts can affect the microbiome, immunity, and physiology.

## 1. Introduction

The first metagenomic studies exploring the phenomenon of colony collapse disorder (CDD) of the economically important western honeybee (*Apis mellifera*) spurred investigations to better understand the bacterial community of the bee gut [1]. Over a decade of research, scientists determined the core bacterial composition of the gut and recognized its role in maintaining honeybee health, while offering a robust parallel to the human microbiota [2]. For example, cumulative results from honeybee and bumblebee feeding studies, whereby bees were fed symbiotic, pathogenic or opportunistic bacteria or pathogenic fungi, revealed consequences of both a stable microbiota and an imbalance of the conventional microbial community [3]. An imbalance of the core constituents of the microbiota is caused by a shift or displacement of these microorganisms by other microorganisms (pathogenic or opportunistic). This is called dysbiosis. Dysbiosis can lead to increased susceptibility to diseases [4] and altered expression of host immunity and physiology genes [5,6]. In the context of CCD, dysbiosis may be one of many confounding factors leading to weakened bee colonies, as bees are unable to cope with external stressors and opportunistic pathogens and diseases [7].

The developed intestine of a worker honeybee gut consists of five core bacterial symbionts that are mainly found in the hindgut: *Snodgrassella alvi*, *Gilliamella apicola*, *Lactobacillus* (Firm-4 and Firm-5), and *Bifidobacterium asteroides* [8]. These constituents are acquired within the home colony and mainly established after nine days of the bee emerging from the cell [9]. Other gut bacteria are frequent, but in relatively lower prevalence and with less consistency across bees and colonies. Many metabolic processes happen in the hindgut, especially in a symbiotic context. Of the core bacterial symbionts, *G. apicola*, *Bifidobacterium*, and *Lactobacillus* (Firm-4 and Firm-5 clades) are carbohydrate fermenters whose metabolic products support *S. alvi* growth. These symbiont-derived metabolites likely also support bee health via intestinal absorption. These findings were supported by microbial feeding studies focused on the physiological processes of bees, and metagenome annotations of the symbionts [6,10]. The midgut lies upstream of the hindgut. The honeybee midgut is the focal point of digestion and absorption of nutrients. It possesses digestive enzymes from the cell lining in addition to enzymes that are secreted and translocated from the hypopharyngeal glands [11,12]. The midgut is also lined with the peritrophic matrix, which is a protective barrier. Relative to the hindgut, the midgut is essentially void of bacteria, possibly due to the constant rearrangement of the peritrophic matrix [9]. The bee midgut is speculated to harbor yeasts that offer additional digestive roles as is common in other insects [13,14]. This indicates that yeasts likely have an alternative method for attachment/colonization in this environment that bacteria lack.

While three pathogenic fungi (*Nosema*, *Ascosphaera apis*, and *Aspergillus*) are well studied [15,16], symbiotic or opportunistic yeasts are generally not a focal point of microbiota research. The honeybee’s intestine is predicted to contain very few yeast and other fungi relative to bacteria, even suggested to constitute less than 1% of the microbiome [17]. Interesting results and ongoing hypotheses from yeast–bee studies have found various interesting peculiarities with respect to gut-residing yeasts. For one, *Nosema ceranae* infections caused an increase of intestinal *Saccharomyces* and *Candida* titers which likely involved excess sugars from *Nosema*-induced malabsorption [17]. Indeed, yeasts are considered general indicators of honeybee stress [18]. However, yeasts may also be beneficial to the insect host. Yeasts seem to offer benefits like the synthesis of vitamins that can supplement bee food [19]. Yeasts also appeared ubiquitous in newly emerged bees and nurse bees and have been speculated to contribute to pollen digestion and royal jelly formulation [20]. Additionally, yeast communities live on flowers and yeasts are collected during foraging. As the foraging process proceeds, the yeast diversity reduces from the collection of pollen to formation and storage of bee bread, suggesting that some fungi are either selected or more suited for the hive environment [21]. The presence of a yeast also increased visiting preferences during pollination [22]. Lastly, colony-level antibiotic treatment will also disturb the yeast community [23].

For this work, we used a midgut-focused, culture-dependent survey on adult honeybees which led to the isolation of *Wickerhamomyces* (previously considered *Pichia* and *Hansenula*) *anomalus* from the honeybee midgut. *Wickerhamomyces,* and more specifically, *W*. *anomalus,* can be found in honeybees as it was identified in many samples of foraging bees over the decades, including this work and current unpublished work [20,21,24]. This yeast is also particularly interesting in mosquitos. The yeast is found in wild mosquitos as well as in decades-old generations of laboratory reared mosquitoes. *Wickerhamomyces anomalus* was found in the mosquito’s intestine and also in the reproductive organs, suggesting a vertical transmission across generations [25,26]. Strains of *W. anomalus* also produce toxins which are broadly antimicrobial [27], and this yeast is also of interest in the food industry and biotechnology [28]. We found that *W. anomalus* can be acquired by honeybees and is a seemingly important constituent of other insects’ microbial communities, possibly having widespread protective, reproductive, and/or digestive roles for insect hosts [14].

Here we describe two separate studies where we investigated the consequences of *W. anomalus* in honeybees (studies A and B). In study A, we sought to explore the effects of yeast augmentation in honeybees that have established bee microbiomes. To this end, we used frame-captured, microbiota-developed honeybees that had no detectable levels of *N. ceranae*. After honeybees were fed the midgut-isolated *W. anomalus* strain, we used a qPCR (quantitative polymerase chain reaction) to monitor transcript changes in the bees’ immunity and physiology, and titers of its established core bacterial microbiota. In study B, newly emerged bees (NEWs: <1-day old and void of an established microbiota; [4]) were first conditioned with *W. anomalus* similar to study A but were then exposed to the virulent microsporidian fungal pathogen *N. ceranae* in order to study possible interorganismal cooperation or competition in the midgut. Although study A was more open-ended from the initial isolation of yeasts and with respect to which effects a yeast will have on the host and bacteria, our hypothesis for study B was that if we pretreated the bee with a putatively symbiotic yeast, then this will alter *Nosema* levels by interfering with its life cycle, perhaps by spatial competition or arguably because certain yeasts like *W. anomalus* have the ability to control its surrounding microbial community through strong antimicrobial secretions. This hypothesis was somewhat similar to another study where honeybees were fed commercialized probiotics with the idea that the probiotics would support honeybee health during a *N. ceranae* infection [29]. In that study, probiotics increased the survival of bees, but did not decrease *N. ceranae* spore counts. Notably, the researchers in that study used a commercial probiotic called Levucell SB^®^ which contains *Saccharomyces cerevisiae boulardii*. In various studies, this yeast can outcompete bacteria and pathogenic yeasts, which suggests that this particular yeast is a promising probiotic yeast. Altogether, we suggest that *W. anomalus* can affect the stable microbiome, alter host immunity— and physiology—related gene expression, and possibly interact with pathogens.

## 2. Materials and Methods

### 2.1. Yeast Isolation, Identification, and Growth Conditions (for Studies A and B)

Honeybees used for yeast discovery and isolation were collected from experimental hives maintained at the USDA Bee Research Lab in Beltsville, MD, USA in October of 2017. These hives were designated “172” and “148”. Honeybees were collected at the entrance of the hives (assuming that the microbial community in the intestine was the most diverse), and four midguts were removed with tweezers. Pooled midguts were macerated by grinding with a sterilized plastic pestle in 400 µL sterile 1× PBS, 100 µL was plated on yeast peptone dextrose (YPD) (bacteriological peptone, 20 g/L; yeast extract, 10 g/L; glucose, 20 g/L; Sigma, St. Louis, MI, USA) amended with agar (2.0% final) and 100 IU Ml^−1^ penicillin—100 μg mL^−1^ streptomycin (5000 IU mL^−1^ and 5000 μg mL^−1^ stock, Cellgro), and incubated at 25 °C under aerobic conditions. Multiple putative yeasts were identified on the agar plate. Various yeasts were isolated by morphology and subsequently restreaked twice on similar plates. To obtain biomass for gDNA extraction and to make glycerol stocks, the isolates were grown in 100 mL YPD broth in 500 mL flasks overnight at 25 °C with agitation (180 rpm). A 700 µL aliquot was combined with 700 µL 50% glycerol in dH_2_0 to make a 25% glycerol frozen stock. A 2 mL aliquot of the overnight culture was pelleted, and the cells were suspended in 200 µL water and then broken by freezing and thawing the suspension three times at −80 °C and 72 °C, respectively. For gDNA isolation, the MagAttract HMW DNA Kit (Qiagen, Hilden, Germany) was used following the manufacturer’s protocol. Primers ITS1_F and ITS4_R (Intergenic regions 1 and 2, and 5.8 s; [30]) and NL1_F and NL4_R (Intergenic region 2 and the large subunit; [31]) were used in a Phusion (NEB) reaction mixture per the manufacturer’s protocol. A cycling PCR was done in a PTC-100 cycler (MJ, Inc.): 98 °C for 30 s, then 40 cycles of 98 °C for 10 s, 55 °C for 15 s, and 72 °C for 60 s, and lastly a 72 °C extension for 10 min. An aliquot of the PCR amplicons was checked in a 2.0% agarose gel for size and singularity, and the remaining amplicons were sequenced. Of the isolates, one fungus was *Wickerhamomyces anomalus*. This was determined from the amplicon sequencing which revealed that the first hit in the NCBI blastn (nr) database for this isolate was *W. anomalus* culture-collection CBS 262 (ITS amplicon: Query cover = 94%, Percent identity = 99.18%, E-value = 0.0, accession hit = KY105853.1) and *W. anomalus* strain S4R1-3 (NL amplicon: Query cover = 96%, Percent identity = 98.79%, E-value = 0.0, accession hit = MG773362.1). Our isolated yeast strain was deposited at the CBS-KNAW, Utrecht, Netherlands (“CBS 15369”). 

For microbe–host feeding experiments, 50 mL of an overnight culture was centrifuged (3000 rcf, 20 °C, up to 10 min), and the pellet was washed with autoclaved dH_2_0 followed by centrifugation and another wash, and finally suspended in 50 mL 1:1 (*v/v*) sucrose–dH_2_0 solution, which was further diluted to an OD_600_ of ca. 0.30 using sucrose–water. Yeasts incubated in the sucrose solution were plated on YPD agar plates to test for viability; colonies of the yeast were recovered on the YPD plate after the yeast was incubated in the sucrose solution for three days. Furthermore, after the yeast incubated in the sucrose solution in a sealed 50 mL tube, the tube became pressurized with bubbles inside, and there was also a strong aroma produced. This suggested that the yeast was fermenting the sucrose and producing volatiles.

### 2.2. Study A

#### 2.2.1. Study A Overview

In study A, we used frame-captured, microbiota-developed honeybees and fed the bees *W. anomalus* to see whether this microorganism will cause significant changes in the bee. To this end, we extracted total RNA from pooled worker bees [32] and then used qPCR to monitor changes in the bees’ immunity, physiology, and core bacterial microbiota. We examined the relative mRNA titers of microorganisms by measuring relative rRNA levels. These included *Lactobacillus* Firm-5, *S. alvi*, *G. apicola*, Universal bacteria, *W. anomalus*, *N. ceranae, and N. apis* (and not by rRNA: Deformed wing virus (DWV)). *Gilliamella apicola*, *Snodgrassella alvi*, and *Lactobacillus* are involved in promoting bee health and in gut metabolism [6]; thus, they are important gut constituents to consider when focusing on the microbiome. DWV (genotype A) was included to determine relative viral loads and apparent bee health, as DWV has a major negative influence on honeybee health and activity, such as colony loss [33,34]. The relative mRNA expression levels from the honeybee host included immunity and physiology targets. To evaluate host immunity, we focused on various genes involved in bacterial, fungal, and viral infections: Antimicrobial peptide effector genes (hymenoptaecin, apidaecin, and abaecin); *AmEater*, an innate defense system gene for phagocytosis; one gene involved in the Toll pathway (Gram-negative bacteria-binding protein; *GNBP1-1*), and two genes involved in the Imd pathway (Peptidoglycan recognition protein; *PGRP-LC* and *PGRP-S1*) [35]. To evaluate effects on physiology, we focused on hormones related to weight maintenance/nutrient homeostasis, which are also influenced by the core microbiota bacteria [6]. These included two honeybee insulin-like peptide genes (*AmILP* and *AmILP2*) and their putative respective receptor genes (*AmINR1* and *AmINR2*). We also examined a general marker gene for bee health (bee host gene: Vitellogenin; Vg); a honeybee putative chitin-binding protein gene that is not exclusively, but more than likely, involved in forming the peritrophic matrix in the midgut (a protective barrier against abrasive food and potential pathogens; [36,37]), and lastly, a honeybee detoxification gene (cytochrome P450_9Q1; [38]).

#### 2.2.2. Bee Collection and Feeding Experiment

Adult honeybees of variable ages (“frame-captured bees”) were collected twice in November 2017 from hive “172” and in February 2018 from hive “5.” Honeybees on frames (variable number) were brushed into a container, the container sealed and then we transported it to the laboratory. Honeybees were immobilized by carbon dioxide, and 30–45 bees were partitioned into clear plastic Solo cups with a plastic lid, similar to [39]. Bees were sorted by pouring the bees from cup to cup. The bulb of a plastic Pasteur pipette was removed, and the bulb was filled with 4.5 mL of feeding solution. The bulb was then inserted into the lid’s straw opening, which provided the bees food and water for the duration of the experiment. Bees were incubated at circa 34 °C with 30%–50% humidity. Mites were present on some bees that were taken from each colony, and these bees were nonetheless used for the experiment (i.e., these bees were randomly pooled with other bees and reared in the bee cups, pooled for genetic extraction, and included in the analyses). For the treatments, from the start some frame-captured bees were fed a 1:1 sucrose–dH_2_0 (*v/v*) solution (control group) and some were fed the yeast–sucrose solution (described in Section 2.1.) (experimental group). Bees were fed *ad libitum* with each cup receiving the same initial amount of feed solution. After three days, the feeders were replaced with fresh solutions of the appropriate food. Feed solution was added to the feeders as necessary. After six days, dead bees, which were left in the cups throughout, were removed, and the remaining live bees were frozen at −80 °C until sorting into extraction bags for total RNA isolation. Bee mortality was recorded as deaths by day 6 divided by the total number of bees in the rearing cup. 

#### 2.2.3. Total RNA Extraction, First-Strand cDNA Synthesis, and qPCR

We extracted total RNA following Section 4.3.2 in the BEEBOOK for “Bulk extraction of RNA from 50–100 whole bees using the acid-phenol method” [32], omitting the hot phenol step. We generally pooled 20 worker bees from a cup into mesh extraction bags. All but four bags had 20 bees per extraction, and for those extractions the lysis buffer volume was adjusted accordingly. For these bags, there was one control cup from February that had 15 bees, one control cup from November that had eight bees, and two yeast cups that had 16 or 15 bees; all of these cups were included in the analyses. One pooled extraction from one cup was considered one replicate to rule out any ‘cup effect’ bias. In total, worker bees fed sucrose water (control) and bees fed *W. anomalus* had 21 and 25 replicates, respectively, from three independent trials (trial 1: Five cups each condition; trial 2: Six cups for the control and nine cups for yeast-fed; and trial 3: Ten cups for the control and eleven cups for the yeast-fed). The determined ΔCqs from all samples of all trials were pooled together for statistical analyses.

The resulting pellet from the total RNA extraction was suspended in 50 µL nuclease-free water by incubating the pellet at 55 °C for 10 min. The RNA quality and quantity were checked spectroscopically with a Nanodrop spectrophotometer (ND-8000). We used a well-established, in-house protocol to produce single-stranded complementary DNA (cDNA) [40]. A total of 1.5 µg of total RNA was aliquoted in 96-well plates (sealed using PCR plate seals (Bio-Rad, Hercules, CA, USA)), which was then treated with amplification-grade Dnase I (1 U; Thermo, Waltham, MA, USA) at 37 °C for 60 min (with inactivation at 75 °C for 10 min). This was followed by cDNA synthesis using SuperScript™ II Reverse Transcriptase (100 U; Thermo) at 42 °C for 50 min (with inactivation 75 °C for 15 min) with priming by 100 ng random hexamers and 50 ng Oligo (dT)_12–18_ (both by Thermo). The cDNA was diluted with 130 µL nuclease-free water and then stored at −20 °C or temporarily at 4 °C when constantly used for qPCR to prevent too many freeze–thaw cycles. 

A final volume of 20 µL qPCR reactions were prepared with 1× SsoAdvanced™ Universal SYBR^®^ Green Supermix (amended with nuclease-free water), 200 nM of each primer, and 1 µL of diluted cDNA. Clear Multiplate™ 96-well PCR plates and Microseal ‘B’ were used (Bio-Rad). The reactions were run in duplicate for two of the three trials and in singlets for one trial. Given the stability of our established qPCR method and consciousness of the amount of reagent required, we ultimately chose to include more replicates than technical qPCR replicates as this would give us more confidence in biological phenomena. Reactions were run in an integrated Bio-Rad C1000 thermal cycler-CFX96 real-time system. The following PCR cycling condition was used: 95 °C for 30 s, and 50 cycles of 95 °C for 5 s and 60 °C or 55 °C (primer dependent) for 15 s (data acquisition). Only primers targeting *S. alvi*, *G. apicola* and Firm-5 were run at 55 °C. The cycling was followed by a melt curve analysis starting with an initial denaturation at 95 °C for 10 s, followed by 0.5 °C increments from 60 °C to 95 °C, each held for 5 s (data acquisition). Established primers (synthesized at Invitrogen, Carlsbad, CA, USA) were used in this work (Table 1). 

We developed a primer pair for this work that was species-specific for *W*. *anomalus*. The primer pair was first created using the annotated large subunit ribosomal (rRNA) sequences in NCBI’s Primer-BLAST online tool (checked against the “nr” database; [41]), and further validated with Silva’s TestPrime 1.0 (LSU r132 database, and using 1 mismatch with 5 bases; [42]), allowing for limited mismatches with other yeasts. Primer efficiency and the limit of detection were determined from a dilution series of yeast-fed honeybee cDNA samples (efficiency percentage for the *W. anomalus*-specific primers: 108%). For *W*. *anomalus*-specific amplification, a Cq of 50 was given to runs that produced a high Cq (because of inconsistent amplification that was likely due to a very low copy number) or where there was absolutely no amplification. qPCR amplicons were purified using the Roche High Pure PCR Product Purification Kit and sent for single primer sequencing at Macrogen (Rockville, MD, USA). Generated sequences were manually trimmed and submitted in NCBI’s nucleotide BLAST [43]. Sequence data of the target amplicons matched rDNA *W. anomalus* sequences in the nr database. Ribosomal protein S5 (*RpS5a*) was used as a reference gene and included for every sample in order to calculate the Δ Cq for analyses [4,44]. The Cq values were automatically determined by Bio-Rad software, and putative outliers were not excluded. The cDNA was checked for detectable levels of gDNA by looking for two generated melt curves using an intron-spanning, reference gene primer set (*Arp1*), as in a previous study [4]; a subset of all the samples was tested, and only a single peak was generated. Some primer pairs were added retrospectively and noted as follows: Only one independent trial was used to examine abaecin transcript levels, and two independent trials were used to examine GNBP-1, PGRP-LC, and PGRP-S1 transcript levels.

### 2.3. Study B

#### 2.3.1. Study B Overview

In study B, newly emerged bees were conditioned with *W. anomalus* and then exposed to *N. ceranae.* As in study A, we used qPCR to monitor changes in immunity, physiology, and microorganism titers, following total RNA isolation using a SDS-NaCl total RNA isolation method.

#### 2.3.2. Bee Collection and Feeding Experiment

Newly emerged worker bees (<1 day old) were collected in October 2018 after naturally emerging from brood combs while in an incubator. These rearing conditions were similar to study A. However, carbon dioxide was not used because young bees can be sorted into cups by hand. Mites were also observed on the bees, which were left as is and included in the pooled total RNA extraction and further analyses. Thirty NEWs per cup were fed either: (i) the sucrose–yeast solution for two days, followed by whole cup-feeding of approximately 200,000 *N. ceranae* spores per bee until full consumption within 12 h and then continuing the sucrose–yeast diet for five days (referred to as “both-fed”); (ii) using the same timeline in (i), with only sucrose solution and *N. ceranae* spores; (iii) also using the same timeline in (i), with the sucrose–yeast solution and without *N. ceranae* spores; and (iv) only sucrose solution the entire time (control). 

#### 2.3.3. Total RNA Extraction, First-Strand cDNA Synthesis, and qPCR

As in study A, we did bulk bee, total RNA extractions but adapted the RNASwift protocol [48]. A separate experiment in our lab was done to compare the total RNA isolation method from study B to that from study A; this is described in the Supplement Materials, including the results therefrom (Table S1). Given that we used two different protocols, we did not pool or combine results from study A and study B, and all results are presented independently. For study B, 20 bees were placed into a mesh extraction bag (Bioreba, Reinach, Switzerland) with 20 mL of prewarmed lysis solution (Sodium chloride 0.5 M; SDS 4.0% w/v; nuclease-free water; all molecular grade). The bees were ground by rolling with a rolling pin. A 500 µL aliquot was removed in triplicate into three separate 1.5 mL centrifuge tubes: Two were frozen at −80 °C as backups, and one was further processed. The tube was placed at 80 °C for five minutes, then 250 µL of 5 M NaCl was added and the tube was mixed by inversion ten times. The solution was centrifuged at 10,000 rcf for 2 min at room temperature. The supernatant was transferred into a new tube that already contained 750 µL molecular-grade 2 propanol, which was then mixed by inversion ten times. The mixture was centrifuged and the supernatant removed, to which 1 mL of 75% ethanol was added. The tube was centrifuged similarly, and the supernatant was then removed. The tube was placed at 55 °C for about five to ten minutes to evaporate the residual alcohol, and 50 µL nuclease-free water was added to suspend the pellet while still in the incubator. Quality control, first-strand cDNA synthesis, and qPCR for these samples were processed essentially as in study A except that the Dnase treatment was changed to use Ambion’s TURBO Dnase, and EDTA was replaced with water. In study B, we used formerly published *W. anomalus*-specific primers, Wa-in and Wa-rev [25], (primer efficiency determined in our system = 98%) as well as the *W. anomalus*-specific primer pair developed in this work. At the end of the experiment, each treatment had ten replicates (1 bee rearing cup of pooled bees = 1 replicate), and we processed replicates for analyses as in study A.

### 2.4. Presentation and Statistical Analyses (for Studies A and B)

Relative transcript quantification was calculated by Δ Cq (Cq *_RpS5a_ —* Cq _Target gene_), where this Δ Cq was used for all subsequent calculations and represented relative messenger RNA transcript levels of a particular gene from a cohort of worker bees. A higher number indicated more transcripts. Calculations were done in Excel (Microsoft, Redmond, WA, USA) using exported data from the Bio-Rad software (3.1). To present the gene expression data, a heatmap was generated with heatmaply and its dependencies in R (v3.5.3, MacOS) [49]. We first calculated the median of all Δ Cqs from both treatments for a specific gene (subtrahend), and then the median of that gene target from a specific treatment (minuend). The resulting Δ Cqs in the heatmap reflect the differences between the minuend and subtrahend. These values are presented in a one-way hierarchical clustering heatmap without further normalization or scaling. For statistical significance tests, the Δ Cq values were imported into JMP (v13 and 14; SAS, Cary, NC, USA). Gene sets were first tested for normality. Because not all gene sets were normally distributed, we universally applied more stringent nonparametric tests for all gene targets. Statistically significant changes in gene expression were therefore determined by pairwise nonparametric comparisons using the Wilcoxon test (*p* < 0.05) from aggregated Δ Cq values from all samples of all trials. Statistically significant differences were amended to the heatmap or boxplot (produced in JMP with an asterisk. Spearman rank correlation (r_s_ where *p* < 0.05) was also done and grouped by treatment in JMP. Comparisons between RNA extraction methods used raw Cq values provided by the Bio-Rad software (3.1) and also values from Nanodrop readings, and were presented as the average of the eight replicates with a standard deviation in parenthesis (Table S1 in the Supplement Materials).

## 3. Results

### 3.1. Study A

#### 3.1.1. *W. anomalus* Influenced Host Immunity, Physiology, and Gut Bacteria

Frame-captured bees that were fed a yeast–sugar solution had significantly higher levels of *W. anomalus* compared to those in sugar only controls (*p* < 0.0001). No amplification occurred for bees not fed the yeast using species-specific yeast primers. Neither *N. ceranae* nor *N. apis* were detected from the frame-captured bees. DWV-A titers did not differ between treatments. From the frame-captured bees, there were no statistically significant changes in the titers of each symbiotic bacterium as well as all bacteria between treatments. Spearman correlations between bacteria that were grouped by treatment indicated significant rearrangements (i.e., correlations) between some bacteria in the intestine (Table 2). Here, changes in the relationship of *Lactobacillus* Firm-5 to other bacteria were the most notable with at least a r_s_ > 0.71 in all cases (*p* < 0.0001). This indicated overall strong, positive correlations between some bacteria. 

**Figure 1:**
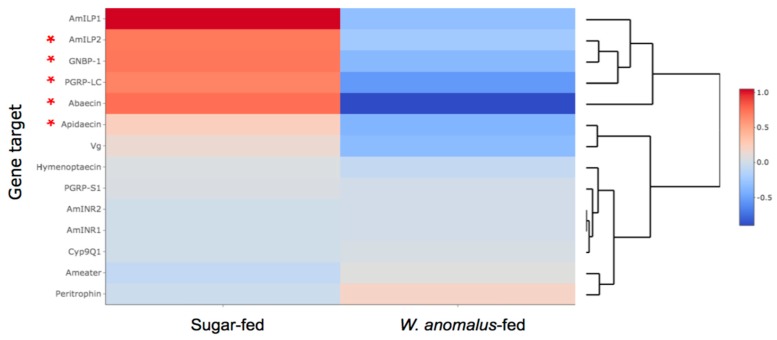
A heatmap amended with a dendrogram (assembled with heatmaply) for study A that shows median normalized, relative gene expression levels (as Δ Cq) of frame-captured bees that were either constantly fed the *W. anomalus*–sucrose solution or only the sucrose solution. Data were pooled from three independent cage feeding trials. The asterisk indicates a statistically significant change in Δ Cq between the treatments of at least *p* < 0.5 by the Wilcoxon pairwise test. Overall, we observed that many immune and hormone-like genes had lower expression after the introduction of *W. anomalus*, which cluster together at the top of the heatmap.

In general, we observed decreases in relative gene expression of immunity-related transcripts when bees were constantly fed *W. anomalus* in comparison to bees fed only sucrose (Figure 1). Statistically significant lower gene expression levels in the *W. anomalus*-fed bees were found for abaecin (*p* = 0.0006), apidaecin (*p* = 0.0127), *PGRP-LC* (*p* = 0.0143), and *GNBP-1* (*p* = 0.0451). Target genes with a lower mean Δ Cq, but not statistically significant, were *PGRP-S1* and hymenoptaecin. There was virtually no change in the mean Δ Cq for *AmEater*. Regarding the insulin-like peptide and receptor genes, the only statistically significant Δ Cq decrease was for *AmILP2* in the *W. anomalus*-fed group (*p* = 0.0099). We observed no statistically significant changes in relative gene expression levels of vitellogenin, peritrophin, or Cyp9Q1.

#### 3.1.2. *W. anomalus* Did Not Increase Short-Term Mortality When Fed to Bees

A one-way ANOVA revealed no statistical difference in honeybee deaths between conditions per trial (*p* > 0.62 in the trials).

### 3.2. Study B

#### NEWs Responded Differently to *W. anomalus*, and *N. ceranae* Affected *W. anomalus*

**Figure 2:**
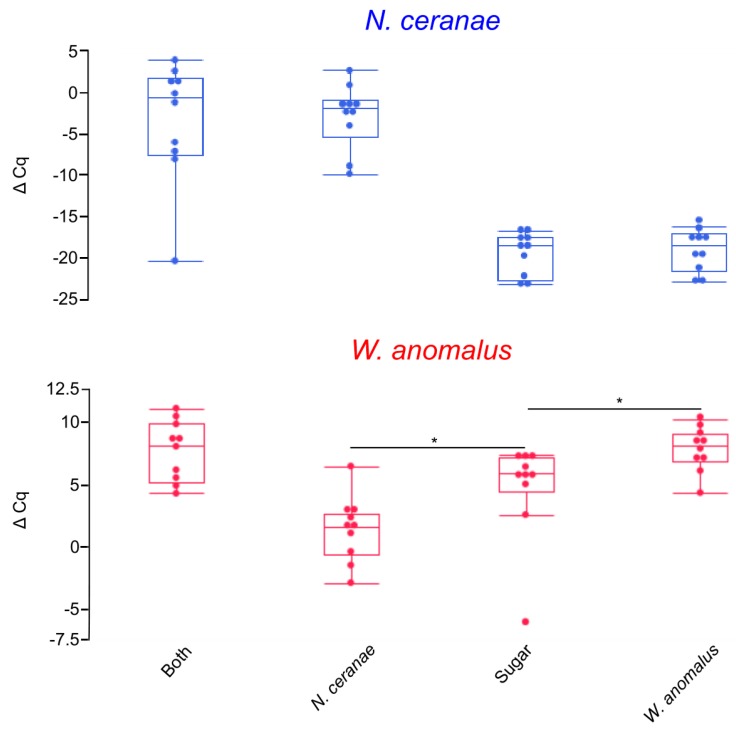
Boxplots for study B showing the relative gene expression values (as Δ Cq) of newly emerged bees that were conditioned with the yeast, followed by feeding *N. ceranae* and then continuous feeding of the yeast (“both”), and respective feeding controls (i.e., *N. ceranae* only, *W. anomalus* only or sugar only). The asterisks indicate statistically significant differences between the treatments of at least *p* < 0.05 by the Wilcoxon pairwise test. The statistical chance for *W. anomalus*-specific targeted qPCR between both-fed and sugar-fed was *p* = 0.0962. Note that when NEWs were not fed *N. ceranae*, there was no amplification of *N. ceranae*, for which a Δ Cq was nonetheless provided (Δ Cq = Cq *_RPS5a_*−50). Overall, we observed that NEWs fed *N. ceranae* had detectable *Nosema* titers which were absent in the NEWs that were not fed *N. ceranae*, and that the naturally occurring *W. anomalus* population decreased after the introduction of *N. ceranae* (sugar vs. *N. ceranae* groups for *W. anomalus*-specific amplification).

Newly emerged bees were first supplemented with *W. anomalus* followed by introduction to *N. ceranae* (“both-fed”) and then a continuation of the *W. anomalus* diet. Controls included replacing the microorganism at each timepoint with sugar water. At the end of the experiment, we observed an increase in mean Δ Cq levels of *W. anomalus* when NEWs were fed *W. anomalus* in comparison to the sugar-fed control (sugar-fed and *W. anomalus*-fed, *p* = 0.0140; sugar-fed and both-fed, *p* = 0.0962; Figure 2). This increase was not as large as in study A where the bees did not contain the yeast prior to artificially feeding the yeast. We tested both our developed primer pair and the formerly published *W. anomalus*-specific primer pair, which both provided comparable results during statistical analyses in JMP; therefore, the previously published primers Wa-in/Wa-rev were presented in Figure 2. In the NEWs, we observed in every case and with two separate *W. anomalus*-specific primer pairs an amplification of *W. anomalus*; therefore, the bees already possessed a naturally occurring baseline level of *W. anomalus* upon naturally emerging from the brood frame. In NEWs with a natural abundance of *W. anomalus* (*N. ceranae*-fed and sugar-fed groups), we observed a statistically significant decrease in relative *W. anomalus* titers when NEWs were fed *N. ceranae* (*p* = 0.0173). *Nosema ceranae* levels were undetectable in NEWs that were not fed *N. ceranae*, whereas in NEWs that were fed *N. ceranae*, we observed significantly higher titers of *N. ceranae* (sugar-fed and *N. ceranae*-fed, *p* = 0.0002; sugar-fed and both-fed, *p* = 0.0013). The samples of NEWs that were not fed *N. ceranae* were given a Cq value of 50 by default. 

After examining microbe titers of *W. anomalus* and *N. ceranae* in NEWs, we followed up with qPCR on a subset of the gene targets from study A which included *PGRP-LC*, vitellogenin, hymenoptaecin, *AmILP2*, and peritrophin in order to cover immunity and physiology (hormonal and structural). For each gene target, we found that there was no statistically significant difference in relative gene expression levels between any of the four treatments. 

## 4. Discussion

Research in the past decade has provided numerous insights into honeybee biology and the microbiota held within their digestive tracts. Most, with the exception of work targeting known fungal and viral pathogens, has focused on the bacterial communities held by honeybees. Here, we veered from the most focused line of fungal research on bees, namely, nosemosis (*Nosema* infection), chalkbrood (caused by *As. apis*) and Stonebrood (*Aspergillus*). This led us to delve deeper into the fungal community (“Mycobiome“) associated with honeybees and its influence on honeybee immunity, physiology, and the bacterial microbiome. 

In honeybees, for example, *Lactobacillales* symbionts are likely players in nutrition homeostasis; cause immunomodulation of the host by stimulating key immune gene expression; and nutritionally and spatially compete with pathogens [50,51]. We had similar hypotheses that the yeast *W. anomalus* may offer similar advantages for the honeybee host as this yeast is particularly known to possess potent antimicrobial effects which may control the surrounding microbes. In the current studies, we investigated the possible effects of the yeast *W. anomalus* on honeybee immunity and physiology; the honeybees’ core microbiota bacterial species; and on the parasitic microsporidian fungus, *N. ceranae*, by molecular profiling. In study A, we observed that in older honeybee hosts, the addition of *W. anomalus* was immunomodulating. This caused generally lower transcript levels of selected immunity genes. Further, after the addition of *W. anomalus*, *Lactobacillus* Firm-5 appeared to have strong, positive correlations with other bacteria. In study B, for newly emerged bees with naturally acquired *W. anomalus*, the introduction of *N. ceranae* caused a decrease in *W. anomalus* titers. Conversely, when NEWs were supplemented with *W. anomalus, N. ceranae* infection did not alter yeast titers. At the moment, it remains unclear how the newly emerged bees picked up such high levels of the yeast, and we speculate that the NEWs may be void of bacteria but may nonetheless carry a relatively low titer of yeast(s) upon naturally emerging from the comb. The yeast then flourishes as the bee ages but is then lost or reduced to undetectable titers as possibly other microorganisms or environmental factors become relevant (e.g., the bacterial microbiota establishes itself, the nutritional role of the yeast is no longer needed or foraging begins). A recent pyrosequencing study targeting not only 16S rRNA genes (bacteria), but also the ITS2 region (fungi) from newly emerged bees to nurse bees to foragers confirmed that the intestinal community extends beyond bacteria and that a fungal community appears to be a common part of the honeybee microbiota [20], confirming previous culture-dependent studies [23,52,53]. Based on our current work, we put forth that honeybee–yeast phenomena are more complex than originally anticipated, and that yeasts may not simply be a sign of weakened or stressed conditions of honeybees [18]. 

As nectar is collected and processed, its complex sugar constituents like sucrose need to be broken down into simpler sugars, like glucose and fructose [54]. This is usually processed by enzymes secreted from the hypopharyngeal glands of the bee that are also passed down to and remain stable within the midgut; enzymes already present in the pollen are also involved [55]. It is possible that floral nectar-residing yeasts participate in the breakdown of sugar while still residing on the flower as well as in the honeybee after nectar collections. In our cup design, the yeast is already present in the sucrose solution and thus arguably changing the chemical composition of the feed by fermenting sucrose into simple sugars, and adding small compounds (i.e., aromates). This controlled feeding design is different than what occurs naturally as we speculate that in floral nectar sugar conversion is slower. Whether predigested sugar truly benefits the honeybee (e.g., bees may now need to invest less energy to secrete digestive enzymes during consumption of carbohydrates) remains to be determined. If yeast fermentation is a positive for sugar digestibility, then this could provide an effective prebiotic for honeybee health.

Yeasts may have a niche in the midgut because most bacteria associated with honeybees do not thrive in this location. Despite the midgut being one of the first destinations of all incoming microorganisms into the intestine and thus a harsh niche to occupy, we believe that one important benefit of residing upstream in the intestinal tract is that the yeasts have the first opportunity to obtain nutrients that are otherwise unavailable to the microbiome. For the host though, this also comes at a cost. It is known that bees absorb simple sugars at the anterior of the midgut. Simply, yeasts that occupy the midgut would be consuming these simple sugars. Therefore, the yeasts could offer some additional benefits to the host in order to occupy the midgut. As studied in other insects, yeasts can offer substantial nutritional benefits to the host, including, but not limited to, various amino acids and vitamin B [56]. *Wickerhamomyces anomalus* also produces chitinases and glucanases that may offer additional degradation of complex compounds or provide protection by antifungal activity (notably, *W. anomalus* is also considered for use as a natural biological control agent; [57,58]). Honey that feeds foragers and the young contains plant-derived antibacterial properties that promote the health of the colony [59,60]. If *W. anomalus* is translocated from nectar to stored food and then into the intestines, the yeast may too offer protective benefits (similar to those plant derivatives) to the colony by protecting food stores or promoting honeybee health in a way that has not been tested in this work, e.g., against bacterial pathogens by limiting nutrients, limiting adherence of pathogens to the host, and/or changing the pH [61]. In fact, even if *W. anomalus* is mildly pathogenic, its putative social passage within the colony may offer superlative benefits within the very complex honeybee superorganism, a phenomenon observed in some ant colonies [62]. Conversely, the yeasts’ occupation in the midgut may be commensal. In this case, occupation may offer no effect on the host. As a final scenario, yeasts may be simply in transit whereby they can be passed onto nestmates and into food, thereby continuing their presence in the hive. Our evidence that bees which fed on the yeast maintained an active (based on yeast transcripts) community of these microbes suggests that *W. anomalus* is an active component of the bee gut. *Nosema* [63] and bacteria [64] are both passed between nestmates by oral transmission (although not exclusively by oral transmission, and also *Nosema* and bacteria that are solely exchanged by oral trophallaxis leads to lower infection prevalence or an atypical microbiota, respectively). Therefore, it is plausible that yeasts can also be exchanged by oral transmission between nestmates, as well as via fecal matter or from nectar, jelly, honey or pollen, and with possibly no benefit to the host.

In our work, we also investigated whether cooperation or competition between *N. ceranae* and *W. anomalus* was occurring, likely in the midgut where each presides. Other work has also focused on yeasts and nosemosis. Researchers found that genera *Candida* and *Saccharomyces* increased over time during nosemosis, but only up until a certain point where then the yeast titers began to decrease; this was only the case during severe, high-dose infections. The researchers speculated that in the beginning of severe nosemosis, nosemosis-induced malabsorption led to excess sugars which benefited yeasts, and after a certain time point, *N. ceranae* finally outcompeted the yeasts. By contrast, lower inoculations of *N. ceranae* led to the proliferation of both organisms for a longer period of time. For our study, we did not find an increase in *W. anomalus* titers, but rather a decrease. This suggested competition as observed in the late stages of nosemosis from the noted study. We speculate that either the dosage of *N. ceranae* changed the dynamics of infection relative to the other study or that *W. anomalus* is just more susceptible to *N. ceranae* in comparison to *Candida* and *Saccharomyces*. 

It is suggested that yeasts play an important role for nurse bees whose role in the hive is to provide food (royal and worker jellies) for the young. Nurses need to consume bee bread (a nutritious food of nectar and pollen) to make the jellies to feed the larvae. The presence of yeasts may help in digestion of pollen and in the detoxification of potential xenobiotics from the plants or other microorganisms [20]. In the pyrosequencing study noted beforehand, researchers found by ITS sequencing that nurse bees possessed only *Saccharomyces*. It was also found that in newly emerged bees and in 12 h old emerged bees, a mycobiome was also present. Here, the mycobiome in newly emerged bees was also exclusively *Saccharomyces* but at a lower abundance compared to nurse bees, and in 12 h old bees, the mycobiome was diverse (but not as diverse as the fungal community in foragers) [20]. Foragers possessed the most diverse fungal community and with almost no *Saccharomyces*, suggesting that newly acquired fungi outcompeted the inceptive fungus of the mycobiome and that *Saccharomyces* may no longer be necessary as it was during younger bees’ development or for their hive duties. On one hand, it is logical to assume that older bees would possess a more diverse and abundant mycobiome because of the constant introduction of (new) microorganisms and the establishment of residents. However, older nurse bees simply contained an almost exclusive yeast population with the largest fungal community compared to all age groups examined. The yeasts probably offer a special role for the specific duties of the nurse bees. Additionally, various microbiological studies have shown that yeasts in acidified, processed pollen appear to be of great value due to their fermentative and nutritional contributions [21]. Conversely, bee bread may possess a very limited fungal community, and nurse bees were then simply only exposed to *Saccharomyces*. However, a recent next-generation RNA sequencing study that included fungi mapping from RNA extracted from worker and royal jellies showed that the fungal community in the jellies is quite diverse and also includes *Saccharomyces*; therefore, we speculate that the produced and also ingested food sources are actually quite “contaminated” with many, diverse metabolically active fungi. This then has a potential for any fungus to colonize the gut in larvae and for cross-contamination to nurse bees [65]. We suggest that these two next-generation sequencing studies argue for the speculated nutritional role of dominating yeasts in nurse bees, but more in-depth research is required to bridge the connection between the yeast community and their putative benefits to the host. 

In connection to our work, we found that older frame honeybees and newly emerged bees responded differently to the introduction of *W. anomalus*. Perhaps the newly emerged bees were more tolerant to the introduction of yeasts in anticipation of establishing a yeast mycobiome, whereas we induced dysbiosis in the older nurse bees by feeding *W. anomalus*. In our work, we also found that the newly emerged bees had already a yeast community, which corroborates that newly emerged bees have a fungal microbiome as noted from the aforementioned next-generation sequencing study. We also observed that the frame-captured bees did not possess the yeast in question; however, we did not check for other fungi or yeast nor did a seasonal survey at our apiary and other apiaries. Using a broader fungal next-generation sequencing study, researchers may be able to monitor the seasonal and age preference of the mycobiome’s constituents and couple this with the role of yeasts in the intestines. We therefore suggest that, in general, yeasts are important during a younger bee’s life, which includes in-house duties to feed the hive, and that the yeast community becomes less essential to the honeybee after foraging begins. One could envisage that yeasts remain in the hive by being an important constituent of nurse bees and passing itself into other bees by remaining in the food sources. 

In study A, we observed that although bacterial titers did not significantly change when bees were fed *W. anomalus*, significant relationships were changed between bacteria, especially *Lactobacillus* Firm-5. Therefore, our data suggest that the change in the amount of each bacteria seems to follow that of *Lactobacillus* Firm-5 when the yeast is present. It was plausible that the yeast created a more acidic environment which was more favorable by acid-tolerant *Lactobacilli* [17]. This dysbiosis by pH may support *Lactobacilli* but will come with additional costs, such as rearrangements of other symbionts and re-enforce nosemosis. Researchers [20] found that *Saccharomyces* was negatively correlated with *Lactobacillus* Firm-5 in newly emerged bees, but *Saccharomyces* was positively correlated with *Lactobacillus* Firm-5 in nurse bees. Our results provided preliminary experimental evidence that a yeast can influence the microbiome. In comparison to our feeding studies, feeding *Lactobacillus* to larvae induced AMP gene expression [50]. Although our study and this study were completely different, one could postulate that *W. anomalus* may not be offering the same probiotic role that *Lactobacillus* offers.

Our studies highlight the need to further explore the effects of dysbiosis in the honeybee intestinal microbiome beyond bacteria, especially since yeasts are likely associated with both negative and positive aspects of every stage of the honeybee’s life. 

## 5. Conclusions

Our results suggest that the yeast *W. anomalus*, isolated from honeybee guts, was immunomodulatory, and its introduction led to lower transcription of selected immune genes in frame-captured nurse bees, which was not the case in very young bees. The parasite *N. ceranae* appeared to influence this gut residing yeast, and likewise, this yeast also seemed to change the relationship between the gut-residing bacteria, especially *Lactobacillus* Firm-5. Overall, we put forth that the honeybee–yeast phenomena remain an intriguing topic with open questions that, when answered, will help us gain an improved holistic understanding of bee microbiota, including both bacteria and yeasts. 

## Figures and Tables

**Table 1:** qPCR primers used in this study, their sequences (5′ to 3′ direction), and a reference.

Target	Forward Primer	Reverse Primer	Ref.
Deformed wing virus (DWV)	GAGATTGAAGCGCATGAACA	TGAATTCAGTGTCGCCCATA	[45]
*Lactobacillus* Firm-5	GGAATACTTCGGTAGGAA	CTTATTTGGTATTAGCACC	[9]
*S. alvi*	CTTAGAGATAGGAGAGTG	TAATGATGGCAACTAATGACAA	[4]
*G. apicola*	GTATCTAATAGGTGCATCAATT	TCCTCTACAATACTCTAGTT	[4]
Universal bacteria	AGAGTTTGATCCTGGCTCAG	CTGCTGCCTCCCGTAGGAGT	[4]
*W. anomalus*	TTTTCGAATCGCATGACTTCGTGTC	GCCTTCCTTGGATGTGGTAGC	[25]
*W. anomalus*	GAGTGAAGCGGCAAAAGCTC	ACAAGAGCCAAACCCAAGGT	This work
*N. ceranae*	TATTGTAGAGAGGTGGGAGATT	GCTATGATCGCTTGCC	[15]
*N. apis*	CTAGTATATTTGAATATTTGTTTACAATGG	GCTATGATCGCTTGCC	[15]
Ribosomal protein (*RPS5a*)	AATTATTTGGTCGCTGGAATTG	TAACGTCCAGCAGAATGTGGTA	[4]
Actin related protein (*Arp1*)	CCAAAGACCCAAGCTCCCTA	TGGCTTATTGGTTTATGTTTTTCGT	[4]
Vitellogenin (*Vg*)	TCGACAACTGCGATCAAAGGA	TGGTCACCGACGATTGGATG	[4]
Insulin-like peptide 1 (*AmILP1*)	CGATAGTCCTGGTCGGTTTG	CAAGCTGAGCATAGCTGCAC	[46]
Insulin-like receptor 1 (*AmILR1*)	GGATCTGGTGTGGGACAGTT	ATCCCCACGTCGAGTATCTG	[46]
Insulin-like peptide 2 (*AmILP2*)	TTCCAGAAATGGAGATGGATG	TAGGAGCGCAACTCCTCTGT	[46]
Insulin-like receptor 2 (*AmILR2*)	GGGAAGAACATCGTGAAGGA	CATCACGAGCAGCGTGTACT	[46]
Apidaecin	TAGTCGCGGTATTTGGGAAT	TTTCACGTGCTTCATATTCTTCA	[44]
Hymenoptaecin	CTCTTCTGTGCCGTTGCATA	GCGTCTCCTGTCATTCCATT	[44]
Abaecin	AGATCTGCACACTCGAGGTCTG	TCGGATTGAATGGTCCCTGA	[47]
Eater	CATTTGCCAACCTGTTTGT	ATCCATTGGTGCAATTTGG	[44]
PGRP-S1	CCCAACAATGCAGCTCTGAA	TTTGGTATTGGTTTGGACGTCC	[47]
PGRP-LC	TCGGAGCGAGATAGTGCATT	CCATCTGCGGTTGTCACTTC	[47]
GNBP1-1	CTCGGGGTAGGAGTTGGTG	ACCATTGATCTTTTGCATGCCA	[47]
Peritrophin	GCAAACGAGATTTCAATGGCAATCTTCAG	CACATTGGTAATTGTATAGTACGTTCGCATC	[37]
Cytochrome P450 (*CYP9Q1*)	ATCCTGGCCAAGTGCAGCTTC	CAGCTCCTTCAATTGGATCAGCAAC	[37]

**Table 2:** Spearman rank correlation (r_s_ where *p* < 0.05 is noted *) of sugar-fed bees and *W. anomalus*-fed bees from study A to examine the relationship between the bacterial microbiota when the yeast *W. anomalus* was and was not present.

**Control**			
**Variable**	**by Variable**	**Spearman r_s_**	**Prob > |r_s_|**
Firm-5	UnvBacteria	0.6844	0.0006 *
*G. apicola*	UnvBacteria	0.606	0.0036 *
*G. apicola*	Firm-5	0.2891	0.2038
*S. alvi*	UnvBacteria	0.6727	0.0008 *
*S. alvi*	Firm-5	0.2714	0.234
*S. alvi*	*G. apicola*	0.6853	0.0006 *
***W. anomalus*-fed**			
**Variable**	**by Variable**	**Spearman r_s_**	**Prob > |r_s_|**
Firm-5	UnvBacteria	0.8682	<0.0001 *
*G. apicola*	UnvBacteria	0.8878	<0.0001 *
*G. apicola*	Firm-5	0.87	<0.0001 *
*S. alvi*	UnvBacteria	0.8667	<0.0001 *
*S. alvi*	Firm-5	0.71	<0.0001 *
*S. alvi*	*G. apicola*	0.7003	<0.0001 *

## Supplementary Materials

The following are available online at https://www.mdpi.com/2075-4450/10/9/296/s1, Table S1: Comparison of two different methods to extract total RNA from pooled, whole honey bees. One method is a modified RNAswift method, and the other uses a homemade lysis solution (COLOSS BEEBOOK (Section 4.3.2.)). The average and its standard deviation from eight replicates per extraction method are shown for Nanodrop-8000 (Thermo) readings (quantity and quality) and qPCR (raw Cqs). RNA integrity was characterized from 2100 Agilent Bioanalyzer runs using a subset of samples and manually inspected.
