# Supplementary material for: Effects of a Resident Yeast from the Honeybee Gut on Immunity, Microbiota, and Nosema Disease
Source: Insects. 2019 Sep 13;10(9):296. doi: 10.3390/insects10090296 (PMC6780889; doi:10.3390/insects10090296)
Supplement: Supplementary file 1 [file insects-10-00296-s001.pdf]

Comparison of total RNA extraction methods

We did a comparison of the two distinct RNA isolation methods from both study A and B. For bulk extractions we compared the RNAswift-based method (methodology within study B) to the COLOSS BEEBOOK protocol "4.3.2. 50-100 whole bees using the acid-phenol method" (excluding the phenol heating step) method, as used within study A. Each method had eight replicates whereby one replicate was a pool of bees from one cup as in study B. Total RNA was analyzed using the 2100 Bioanalyzer Instrument (Agilent) for "Eukaryota Total RNA Nano" and a Nanodrop spectrophotometer (ND-8000). First-strand cDNA synthesis and qPCR was done like in study B, but notably a fixed volume of variable µg total RNA (in all cases at least 1.6 µg) was used in the DNase treatment / cDNA synthesis reaction. *Rps5a*, vitellogenin (*Vg*) and DWV-A were checked by qPCR (as executed in study A) and compared to a positive control. A no reverse transcriptase control was done to detect DNA contamination in all samples, and a NTC control was performed in each qPCR plate to check for contamination and dimers. Comparison of the two total RNA extraction methods used in this work are provided here (Table S1).

Comparable RNA extraction methods

The RNAswift-based protocol is a student-friendly (*i.e.*, ease, simplicity and lack of toxic reagents) total RNA extraction method, which only needs salt, SDS, water and alcohol. Various benefits include safer working conditions for the scientist, improved access for student projects and labs with limited resources and access to chemicals, reduced hazardous wastes, reduced cost, and faster preparation time.

For each method, each total RNA extract suspended in molecular-grade water was measured by Nanodrop for quantity and initial quality, and a randomly selected subset of the total RNA extracts was measured using a Bioanalyzer to measure RNA integrity, followed by qPCR after first-strand cDNA synthesis.

In regards to the Nanodrop readings, the RNAswift-based method had a notably smaller concentration of total RNA extracted yet a higher $A_{260/230}$ in comparison to the BEEBOOK method. Both methods had the desirable $A_{260/280}$ around 2.0. For the integrity analyses, a ribosomal RNA ratio number could not be determined as expected for heat-treated honeybee RNA which produces only a single peak, thus electropherograms were individually inspected for a large single peak (18s + 28s combined) and an ideal flat baseline. In regards to the BEEBOOK method, we observed a single large peak and a flat baseline. For the RNAswift-based method, we observed in all but one case a single large peak, but with a slightly higher baseline which indicated the start of RNA degradation.

qPCR, the end reaction goal of each extraction method, was performed targeting a honeybee reference gene (*RPS5*), a general health marker gene (vitellogenin, *Vg*) and Deformed wing virus (DWV-A). We collected and averaged raw Cq values from eight replicates. Both RNAswift and the BEEBOOK methods showed acceptable amplification with consistent results between extraction methods. The averaged raw Cq values per method (with the standard deviation in parentheses) and the quantity, quality and integrity of the total RNA are shown in Table S1. We found that despite the initial indication that RNA was degrading, the endpoint goal of qPCR provided solid runs and Cq values that were compatible between methods.

Table S1. Comparison of two different methods to extract total RNA from pooled, whole honeybees. One method is a modified RNAswift method, and the other uses a homemade lysis solution (COLOSS BEEBOOK (section 4.3.2.)). The average and its standard deviation from eight replicates per extraction method are shown for Nanodrop-8000 (Thermo) readings (quantity and quality) and qPCR (raw Cqs). RNA integrity was characterized from 2100 Agilent Bioanalyzer runs using a subset of samples and manually inspected.

**Method**	**Type**	**ng/μl**	**260/280**	**260/230**	***RpS5* Cq**	***Vg* Cq**	**DWV Cq**	**RNA integrity**
**RNAswift**	**Bag**	370.71(236.97)	2.07(0.12)	1.78(0.41)	23.41(1.25)	22.28(2.05)	29.09(5.1)	Initial degradation
**Homemade**	**Bag**	1209.79(235.73)	2.07(0.08)	1.38(0.09)	25.78(4.14)	27.38(4.79)	25.08(5.04)	Intact
**Positive**	**-**	N/A	N/A	N/A	25.03	27.42	9.33	N/A
